# Evolution of unusual morphologies in Lentibulariaceae (bladderworts and allies) and Podostemaceae (river-weeds): a pictorial report at the interface of developmental biology and morphological diversification

**DOI:** 10.1093/aob/mcv172

**Published:** 2015-11-20

**Authors:** Rolf Rutishauser

**Affiliations:** Institute of Systematic Botany, University of Zurich, Zurich, Switzerland

**Keywords:** Deconstrained bauplans, developmental robustness, evolutionary developmental biology, hopeful monsters, molecular genetics, morphospace, process morphology, root–shoot bauplan, saltational evolution, *Dalzellia*, *Genlisea*, *Tristicha*.

## Abstract

**Background** Various groups of flowering plants reveal profound (‘saltational’) changes of their bauplans (architectural rules) as compared with related taxa. These plants are known as morphological misfits that appear as rather large morphological deviations from the norm. Some of them emerged as morphological key innovations (perhaps ‘hopeful monsters’) that gave rise to new evolutionary lines of organisms, based on (major) genetic changes.

**Scope** This pictorial report places emphasis on released bauplans as typical for bladderworts (*Utricularia*, approx. 230 secies, Lentibulariaceae) and river-weeds (Podostemaceae, three subfamilies, approx. 54 genera, approx. 310 species). Bladderworts (*Utricularia*) are carnivorous, possessing sucking traps. They live as submerged aquatics (except for their flowers), as humid terrestrials or as epiphytes. Most Podostemaceae are restricted to rocks in tropical river-rapids and waterfalls. They survive as submerged haptophytes in these extreme habitats during the rainy season, emerging with their flowers afterwards. The recent scientific progress in developmental biology and evolutionary history of both Lentibulariaceae and Podostemaceae is summarized.

**Conclusions** Lentibulariaceae and Podostemaceae follow structural rules that are different from but related to those of more typical flowering plants. The roots, stems and leaves – as still distinguishable in related flowering plants – are blurred (‘fuzzy’). However, both families have stable floral bauplans. The developmental switches to unusual vegetative morphologies facilitated rather than prevented the evolution of species diversity in both families. The lack of one-to-one correspondence between structural categories and gene expression may have arisen from the re-use of existing genetic resources in novel contexts. Understanding what developmental patterns are followed in Lentibulariaceae and Podostemaceae is a necessary prerequisite to discover the genetic alterations that led to the evolution of these atypical plants. Future molecular genetic work on morphological misfits such as bladderworts and river-weeds will provide insight into developmental and evolutionary aspects of more typical vascular plants.

## INTRODUCTION

### Complementarity in describing living organisms

‘It should be observed that there is no language, so no thought whatever, and no science, without typology’ (Guédès, 1979, p. 17). Thus, natural sciences usually require a clear-cut language consisting of well-defined terms and notions that allow either–or decisions. However, drastic evolutionary changes in bauplans of living organisms may require fuzzy rather than clear-cut concepts of organ identity for description (Rutishauser and Isler, 2001; Kirchoff *et al.*, 2008; Rutishauser *et al.*, 2008; Wang *et al.*, 2011; Minelli, 2015*a*, *b*). Various philosophers and scientists (e.g. Sattler, 1986, 1996; Korzybski, 2010) accepted two or more complementary views, perspectives or modes to describe and interpret form and function of living matter, including growth of plant structures (Sattler and Rutishauser, 1990).

### Bauplans vs. morphological misfits in biology

Groups of related organisms (animals, plants, fungi) usually have a set of architectural rules in common which are called the bauplan (body plan, constructional plan). Bauplan in living organisms captures the idea of the architectural constraints existing in such a functional design. Bauplans are generalizations of our thinking and classifying brain. There is no doubt that certain animals and plants evolved structures (organs, appendages) that cannot be sensibly accommodated in traditional descriptions. Some plant groups were outlined as morphological misfits by Adrian Bell (1991), who highlighted the fact that morphological misfits are ‘misfits to a botanical discipline not misfits for a successful existence’. Morphological misfits are also observable in animals (Minelli, 2015*b*). Various morphological misfits emerged as morphological key innovations (perhaps ‘hopeful monsters’) that gave rise to new evolutionary lines of organisms (Theissen, 2006, 2009; Masel and Siegal, 2009). Morphological misfits provide opportunities for investigating character evolvability. The concept of ‘morphological misfits’ is an eye-catcher that allows labelling of all kinds of morphological deviations in the wild, mainly based on major genetic changes such as homeosis (ectopic gene expression in a seemingly wrong position), and other kinds of developmental repatterning (Arthur, 2011; Minelli, 2015*b*, 2016).

In most seed plants, there is only one major type of construction, the classical root–shoot (CRS) bauplan, with roots and shoots (i.e. stems with leaves) as bauplan units, as well as ‘flowers’ (i.e. unbranched short shoots) serving for sexual reproduction. Strong deviations from the CRS bauplan are usually taken as morphological misfits. Well-known morphological misfits in flowering plants are the Lentibulariaceae (bladderworts and allies) and the Podostemaceae (river-weeds). Both families have members with released (decanalized) body plans, strongly deviating from the CRS bauplan of typical seed plants. The change from terrestrial life to the aquatic habitat may have caused the loss of the CRS bauplan. This seems to be the case in Podostemaceae; less so in Lentibulariaceae (as will be discussed below). Bell (1991) described the free-aquatic duckweeds (*Lemna* and allies, Araceae) with thalloid stem–leaves and the one-leaf plants (*Monophyllaea*, *Streptocarpus*, Gesneriaceae) as additional examples of morphological misfits in flowering plants (Landolt, 1998; Möller and Cronk, 2001; Ayano *et al.*, 2005; Harrison *et al.*, 2005; Cusimano *et al.*, 2011; Tsukaya, 2014).

### Aim of this paper

**Fig. 1.**
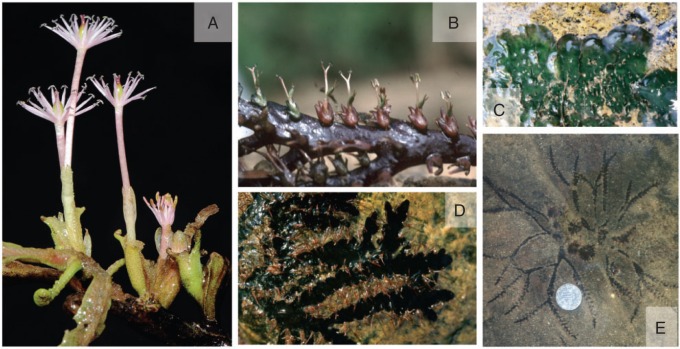
Podostemoid river-weeds (Podostemaceae – Podostemoideae), as observable in nature. (A) *Apinagia latifolia* (K.I.Goebel) P.Royen [Source: Bittrich & Amaral; Brazil, Serra do Tepequem] with showy flowers pollinated by insects. (B) *Polypleurum dichotomum* (Tul.) Cusset [Source: Rutish. & Huber; India, Kerala]: inconspicuous wind-pollinated flowers, arising from endogenous buds of free-floating root. (C) *Hydrobryum japonicum* Imamura *s.l.* [Source: Rutish.; Japan, Kyushu]: crustose green root, firmly attached to rock, resembling foliose lichen. (D and E) *Griffithella hookeriana* (Tul.) Cusset [Source: Rutish. & Huber; India, Kerala]: broad and narrow ribbon-like roots, attached to rock, an example of intraspecific variation.

**Fig. 2.**
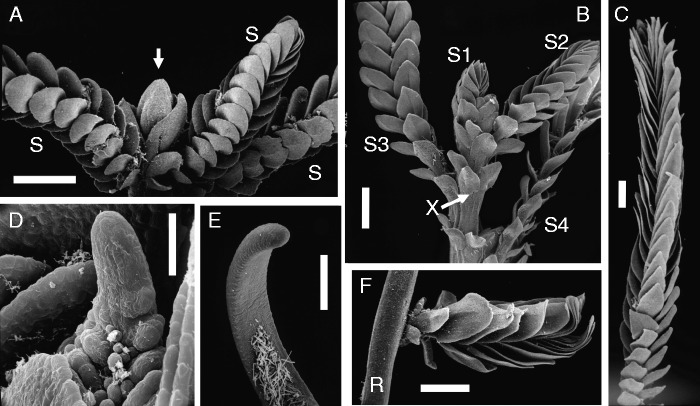
Basal members of tristichoid river-weeds. (A–E) *Tristicha trifaria* (Bory ex Willd.) Spreng. [Novelo & Philbrick s.n. March 1992: Mexico, Jalisco]. (A) Floral shoot with terminal flower (arrow) and three photosynthetic shootlets (S), called ‘ramuli’, with scale-like leaves along three rows. (B) Tip region of 12 mm long vegetative shoot with four ramuli (S1–S4). Note additional scale-like leaves inserted along stem (X). (C) Upper portion of fully grown ramulus (total length 3 cm). (D) Lateral view of meristematic ramulus tip (slightly curved). (E) Ribbon-like root with capless tip, seen from below. Note presence of adhesive hairs (‘root hairs’) on lower surface. (F) *Terniopsis malayana* (Dransfield & Whitmore) M.Kato [Dransfield KEW#30762: Malaysia, Malaya]: Creeping root (R), seen from above, with young ramulus, showing scale-like leaves in three rows. Scale bars = 1 mm in A, B, C–F; 0·05 mm in D.

**Fig. 3.**
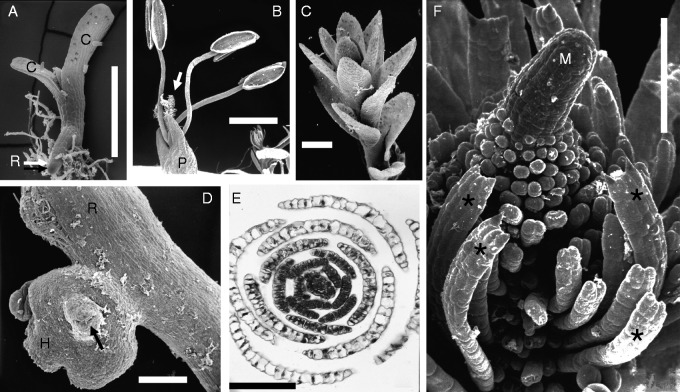
Tristichoid river-weed *Indotristicha ramosissima* (Wight) van Royen [Rutish. & Huber #27/185: India, Kerala]. (A) Seedling with two cotyledons (C) and short-lived plumule, adventitious root (R) as exogenous outgrowth of hypocotyl. Note adhesive hairs replacing radicle. (B) Flower in anthesis, perianth (P) overtopped by three stamens and stigma (arrow). (C) Tip of nearly mature ramulus (total length 12 mm), showing scale-like leaves in helical arrangement. (D) Portion of creeping, ribbon-like root (R), seen from above. Note endogenous origin of disk-like holdfast (H), fixing the shoot bud (black arrow) to the rock. (E) Transversal section of growing ramulus tip. Note spiral arrangement of broad scale-like leaves, consisting of a single cell layer each. (F) Meristematic tip of young ramulus giving rise to ligulate leaves (asterisks). Apical meristem (M) conical and slightly curved. Scale bars = 1 mm in B, C, E; 0·5 mm in A, D; 0·1 mm in F.

**Fig. 4.**
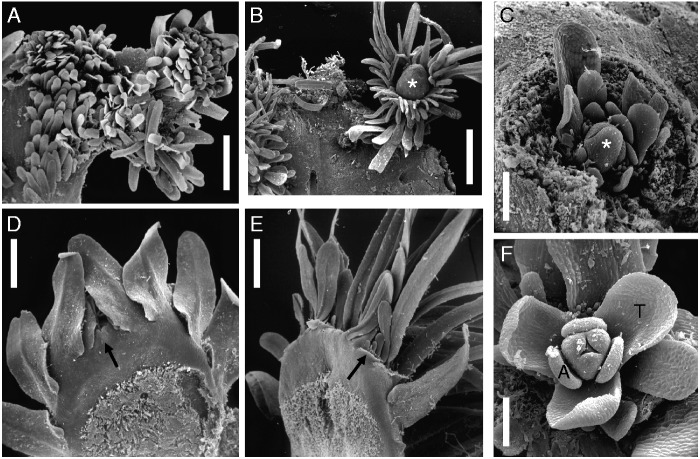
Tristichoid river-weed *Dalzellia zeylanica* (Gardner) Wight [Rutish. & Huber #25/181: India, Kerala]. (A) Crustose creeping shoot (resembling foliose lichen) in vegetative stage, as seen from above; scale-like leaves inserted on upper surface and along margin. (B) Mature stage of crustose creeping shoot, as seen from above; most scale-like leaves dropped. Note reproductive short shoot with floral bud (asterisk), embedded in a fringed cup (cupule). (C) Young short shoot with floral bud (asterisk), showing endogenous origin in cortical tissue of crustose shoot. (D and E) Marginal portion of two young crustose shoots, as seen from below. Arrow points to ‘shoot meristems’ where new marginal leaves are initiated. Note scale-like leaves (with midrib) of variable shape. (F) Flower (prior to anthesis) with three tepals (T), three stamens (A), trimerous ovary. Scale bars = 1 mm in A, B; 0·3 mm in C–E; 0·2 mm in F.

**Fig. 5.**
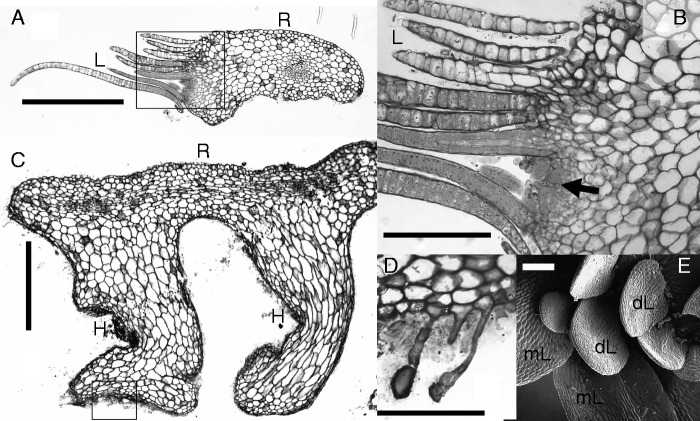
Tristichoid river-weed *Indodalzellia gracilis* (Mathew, Jäger-Zürn & Nileena) Koi & M. Kato [C.R.Mathew #MRPII/430&II/470: India, Kerala]. (A) Cross-section of ribbon-like root (R), giving rise to root-borne crustose shoot on the left flank. (B) Close-up (see insert in A); arrow points to shoot meristem. Note scale-like leaves (L) that mainly consist of one cell layer. (C) Cross-section of root (R) with two finger-like holdfasts (H), growing downwards to reach the substrate. (D) Close-up of holdfast epidermis (see insert in C), showing adhesive hairs. (E) Close-up of crustose shoot, seen from above; dorsal leaves (dL) smaller than marginal leaves (mL). Scale bars = 0·5 mm in A,C; 0·1 mm in B, D, E.

**Fig. 6.**
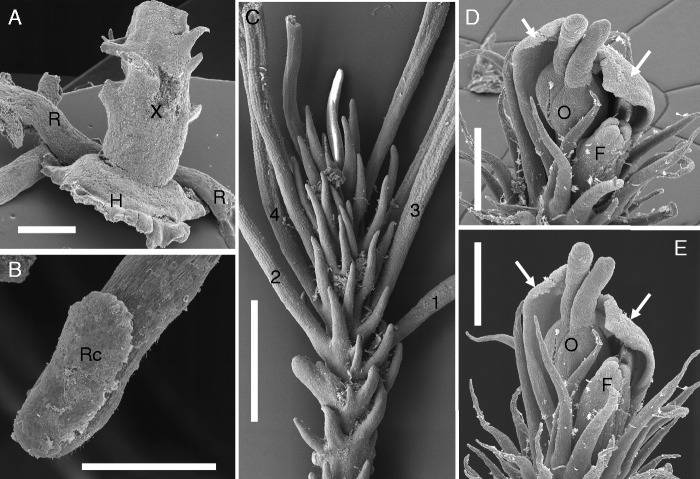
Brazilian podostemoid river-weed *Diamantina lombardii* Novelo, Philbrick & Irgang [Philbrick #5647/5783: Brazil, Rio do Peixe near Diamantina]. (A) Ribbon-like creeping root (R) giving rise to stem (X) with disk-like holdfast (H). (B) Ribbon-like root with capped tip (Rc). (C) Upper portion of vegetative shoot, digitate leaves, each with elongate middle finger and much shorter lateral ones. Numerals 1–4 indicate distichous phyllotaxis. (D and E) Two views of shoot tip with two flowers. Sub-terminal flower with ovary (O) and two stigmas, subtended by open bract (arrows). Terminal flower (F) covered by tubular spathella. Scale bars 1 mm in A, C; 0·5 mm in B, D, E.

**Fig. 7.**
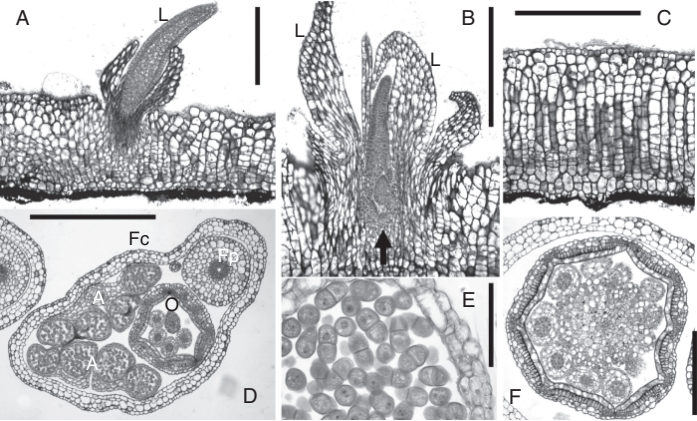
African podostemoid river-weed *Ledermanniella linearifolia* Engl. [Ghogue #1415: Cameroon, Lobé Falls]. (A–C) Transversal sections of crustose roots, with endogenous root-borne shoots (carrying leaves L) arising from upper surface; arrow in close-up (B) indicates position of indistinct shoot meristem. Cell rows inside crustose roots result from thickening growth. Note remnants of adhesive layer (black) on lower root surface. (D–F) Cross-sections of floral bud inside spathella (Fc), with two stamens (A), hanging ovary (O) and pedicel (Fp). Details: (E) anther with pollen dyads; (F) non-septate ovary with ovules on central placenta. Scale bars = 0·5 mm in A–D, F; 0·1 mm in E.

**Fig. 8.**
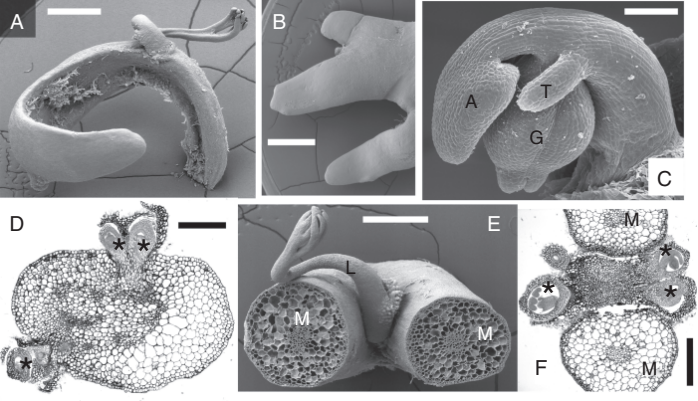
African podostemoid river-weed *Stonesia ghoguei* E. Pfeifer & Rutish. [Ghogue #1665/1668: Cameroon, Adamaoua]. (A) Tip of ribbon-like root, lacking root cap. Note adhesive hairs on lower (inner) root surface, and root-borne shoot with forked leaf. (B) Tips of exogenously branching root ribbon, seen from above. (C) Inverted flower bud (spathella removed), with stamen (A), ovary (G), tepal (T). (D) Transversal stem section, with two lateral short shoots containing floral buds (asterisks). (E and F) Leaf (L) and flowers (asterisks) as epiphyllous outgrowths, arising from angles of forked mother leaf (M). Scale bars = 1 mm in A, B, D–F; 0·5 mm in C.

**Fig. 9.**
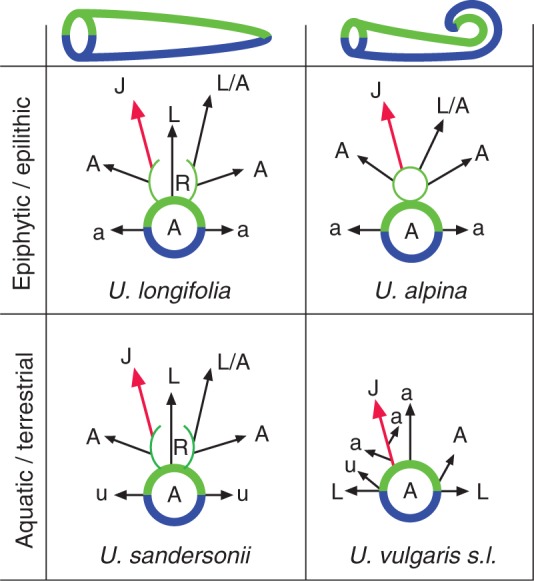
Schemes of stolon branching in various bladderworts (*Utricularia*), showing dorsiventral symmetry. Mother stolons (runners) with dorsiventral branching pattern: green = upper stolon sector, blue = lower sector. Stolon tips (apical meristems) straight or coiled (‘circinate’), depending on the subgenus (and section) in *Utricularia*: *U. alpina* (sect. *Orchidioides*) as epiphytic member, *U. longifolia* (sect. *Foliosa*) as epilithic member, *U. sandersonii* (sect. *Calpidisca*) as terrestrial member of subgenus *Bivalvaria*, *U. vulgaris s.l.* (sect. *Utricularia*) as aquatic member of *Utricularia* subgenus *Utricularia*. Stolon outgrowths (as seen from distal end) are abbreviated as follows: R, rosette of various appendages; L, leaf; A and a, thick and thin daughter stolons; L/A, daughter stolon and leaf arise from same position (homotopic); u, trap (bladder); J (red arrow), inflorescence. All outgrowths (appendages) are inserted along dorsal (green) and lateral stolon sectors, none along ventral (blue) sector. Rosettes in *U. longifolia* and *U. sandersonii* are inserted in proximal (‘wrong’) axil of foliage leaf (L) along dorsal sector of mother stolon (A).

**Fig. 10.**
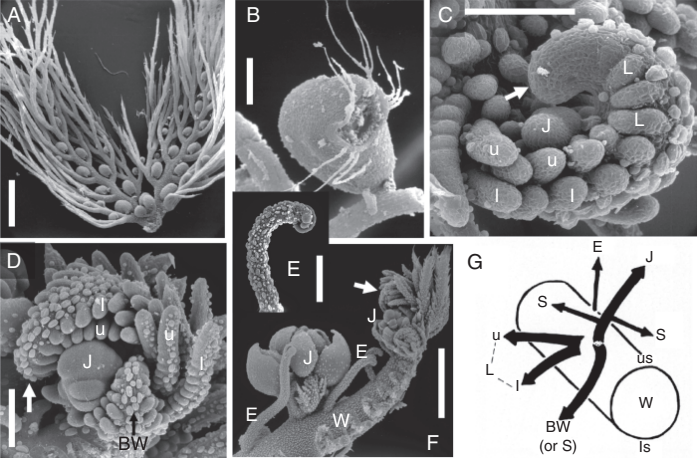
Aquatic bladderworts (section *Utricularia* in subgenus *Utricularia*): (A and B) *Utricularia australis* R.Br. [Rutish. #88710: Switzerland]. (A) Compound leaf with two branched lobes (immature), both with many traps. (B) Mature trap with lateral mouth, two dorsal appendages (branched) and lateral setae (simple). (C–F) *Utricularia aurea* Lour. [Rutish. & Huber #8907036: India, Kerala]. (C and D) Coiled (‘ciricinate’) meristematic tips (white arrows) of stolons (watershoots), with lateral insertion of bilobed leaf primordia (L), each with upper (u) and lower (l) lobe; inflorescence bud (J) arising from upper stolon sector. (E) Curved tip of air-shoot. (F) Distal portion of stolon (W, watershoot) after removal of leaves; inflorescence buds (J) and air-shoots (E) inserted along upper (dorsal) stolon sector. (G) Branching scheme, as valid for many aquatic bladderworts (section *Utricularia*): stolon portion of watershoot (W, seen from distal end), showing dorsiventral symmetry (us, upper sector; ls, lower sector); laterally inserted leaf (L) with upper (u) and lower (l) lobe; inflorescence (J), accompanied by branch watershoot (BW) and anchor stolons (S; ‘rhizoids’ or ‘floats’); extra-axillary air-shoot (E) inserted along dorsal stolon sector. See Fig. 9 (bottom right) for a more generalized branching scheme of *U. vulgaris s.l.* Scale bars = 1 mm in A, F; 0·3 mm in B, D, E; 0·1 mm in C.

**Fig. 11.**
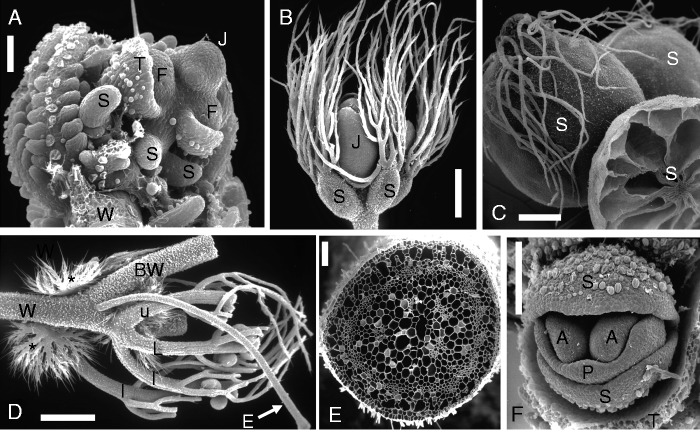
Float-bearing aquatic bladderwort *Utricularia stellaris* Linn.f. [CDK Cook s.n.: India, Rajastan]. (A) Tip of stolon (W, watershoot) with inflorescence apex (J), floral meristems (F) subtended by bracts (T); S, float primordia (replacing anchor stolons). (B and C) Immature and (nearly) mature whorls of spongy floats (S), respectively; note aerenchyma inside. (D) Portion of stolon (watershoot) with branch watershoot (BW). Asterisks indicate stipule-like auricles. Abbreviations of other appendages as in Fig. 10. (E) Cross-section of inflorescence axis (peduncle), showing vascular ring. (F) Young flower, subtended by bract (T); with two sepals (S), two stamens (A) and lower corolla lip (P, upper lip hidden). Scale bars = 1 mm in B–D; 0·1 mm in A, E, F.

**Fig. 12:**
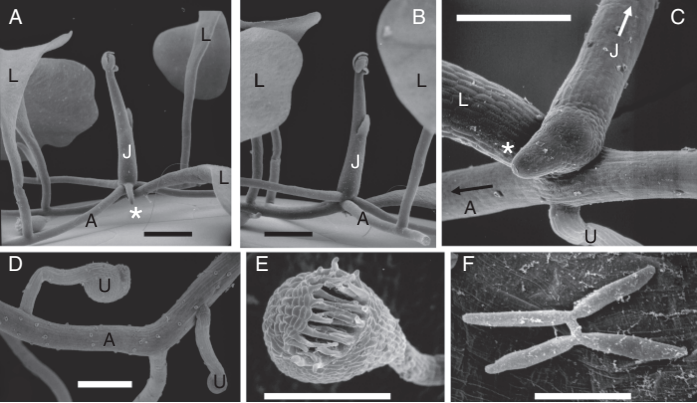
Terrestrial bladderwort *Utricularia sandersonii* Oliv. [cultivated material: Bot. Garden Zurich]. (A and B) Two views of same plant portion, with young inflorescence axis (J), capillary runner stolons (A) giving rise to petiolate leaves (L) with cuneate blades. Asterisk indicates young daughter stolon. (C) Close-up of young rosette inserted in proximal (‘wrong’) axil of foliage leaf (L) along dorsal sector of runner stolon (A). Arrows indicate growth direction of both runner stolon and inflorescence (J) axis. Asterisk indicates primordial daughter stolon. Note trap stalk (U) as lateral stolon appendage. (D) Stolon (A) carrying stalked young traps (U). (E) Nearly mature trap with stipitate glands around terminal mouth. (F) Quadrifid gland inside trap. Scale bars = 1 mm in A, B; 0·5 mm in C–E; 0·1 mm in F.

**Fig. 13:**
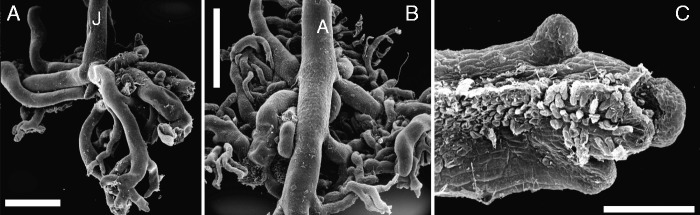
Rheophytic bladderwort *Utricularia neottioides* A.St.Hilaire & Girard [Vogel s.n.: Brazil. (A) Basal portion of inflorescence stalk (J) with claw-like anchor stolons (‘rhizoids’) fixing plant to rock. (B) Another portion with creeping main stolon (A) giving rise to minor claw-like stolons. (C) Close-up of branched tip of claw-like anchor stolon, seen from below. Note adhesive hairs. Scale bars 1 mm in A, B; 0·1 mm in C.

**Fig. 14:**
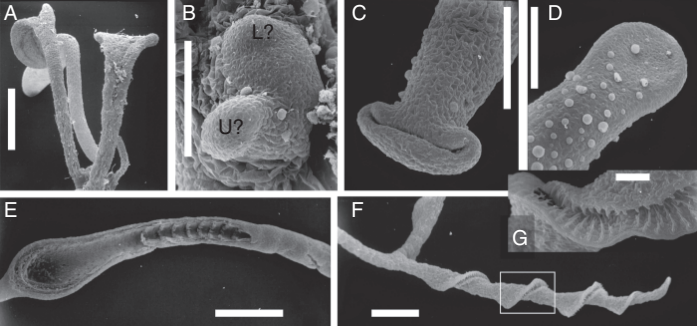
Corkscrew plant *Genlisea repens* Benj. [Bütschi s.n.: Venezuela, Auyan Tepui]. (A) Seedling with rosette of green leaves. (B) Shoot meristem of seedling with putative leaf primordium (L?) and putative eel trap primordium (U?). (C) Peltate (ascidiate) eel trap primordium with transversal slit. (D) Young foliage leaf with spoon-like blade. (E) Proximal portion of nearly mature eel trap (‘rhizophyll’), artificially opened. Note bulb (with digestive glands) and tube (with bristles inside, arranged in rings). (F) Proximal portion of nearly mature eel trap (‘rhizophyll’), showing one of the two twisted arms (corkscrews) with helical slit. (G) Close-up of bristly slit (see insert in F), as entrance path for prey. Scale bars = 1 mm in A, E, F; 0·1 mm in B, C, D, G.

There is no consensus of opinion on how to label and to describe the various structural units comprising the vegetative bodies in both Lentibulariaceae and Podostemaceae. Examples for these terminological difficulties will be given below under the headings ‘The river-weed puzzle’ (Figs 1–8) and ‘The bladderwort puzzle’ (Figs 9–14). It will be shown that Lentibulariaceae and Podostemaceae (in spite of being labelled as morphological misfits) have unique sets of architectural rules (branching patterns) that may be called ‘bauplans’ again. In the final discussion, the findings on bladderworts and river-weeds are incorporated into a more general concept on developmental robustness. The question remains as to whether or not the developmental switches to rather unusual new bauplans had facilitated rather than prevented the evolution of species diversity across bladderworts and river-weeds. The present essay places emphasis on the heuristic value of Sattler’s continuum approach and fuzzy ‘Arberian’ morphology for developmental genetics and character evolution transgressing plant organs, e.g. root–shoot indistinction (Arber, 1920, 1950; Sattler, 1986, 1996; Rutishauser, 1995, Rutishauser and Isler, 2001; Kirchoff *et al.*, 2008; Rutishauser *et al.*, 2008). All scanning electron micrographs (SEMs) and microtome sections (Figs 1–14) were produced in the author’s lab at Zurich University with material collected in the field (see figure legends for details on specimens). The material and methods used have been described elsewhere in detail (e.g. Rutishauser, 1997; Rutishauser and Isler, 2001; Ameka *et al*., 2003; Moline *et al*., 2007).

### Recognition of genera and species in Lentibulariaceae and Podostemaceae

Both families have about the same number of species (slightly more than 300). However, with respect to numbers of genera, they are quite different. The Lentibulariaceae consist of three genera only. With approx. 360 species they are by far the most diverse carnivorous family in flowering plants: approx. 29 *Genlisea* species, approx. 100 *Pinguicula* species and approx. 230 *Utricularia* species are accepted as good species (Taylor, 1989; Fleischmann, 2012*a*, *b*; Veleba *et al.*, 2014). With 53 genera for a total of approx. 310 species, the Podostemaceae (river-weeds) is a rather odd family (Cook and Rutishauser, 2007; Philbrick *et al.*, 2010; Kato, 2013).

## THE RIVER-WEED PUZZLE: THE EVOLUTION OF UNUSUAL MORPHOLOGY IN THE PODOSTEMACEAE (Figs 1–8)

### Adaptation to unique habitats

Podostemaceae is a family of unusual aquatic eudicots within the angiosperms. The plants grow submerged on rocks in apids and waterfalls of clean rivers, mostly in tropical and sub-tropical regions. They survive as submerged haptophytes (and rheophytes) in these extreme habitats during the rainy season. At the end of the rainy season, the water level recedes and the plants emerge, with anthesis usually above the water level. Basal podostemoid members from the Neotropics (e.g. *Mourera*) are known to be visited by insects such as *Trigona* bees (Sobral-Leite *et al.*, 2011). More derived American and all non-American members of Podostemaceae are wind-pollinated or cleistogamous (Philbrick and Les, 1996; Cook and Rutishauser, 2007; Sehgal *et al.*, 2009). All Podostemaceae investigated to date lack double fertilization. Consequently, there is no endosperm (Cook and Rutishauser, 2007; Sehgal *et al.*, 2011). Most Podostemaceae are annuals, dying after having reproduced sexually with minute wind-dispersed seeds. At the beginning of the new rainy period, the seeds stick to submerged rocks (rarely wood or concrete) and germinate into seedlings with adhesive hairs (Fig. 3A). Most Podostemaceae colonize the substrate with prostrate dorsiventrally flattened bodies fixed to the rocky substrate by adhesive hairs and/or finger-like anchoring organs, called ‘holdfasts’ (Figs 2E, 5C, D and 8A). Adhesive hairs are reported to secrete ‘super glue’ (Mohan Ram, 1992, 2001). Moreover, sticky biofilms produced by cyanobacteria help to attach the roots to the rocky substrate (Fig. 7A; Jäger-Zürn and Grubert, 2000; Jäger-Zürn, 2003). Flowering time appears to be critical for Podostemaceae. When the plants emerge, flowers and fruits are produced in a short time. A high flower number is attained by initiating floral buds nearly everywhere on the vegetative body (as will be shown below).

### Molecular systematics

The family is sister to Hypericaceae (within clusioid Malpighiales, eudicots) based on molecular phylogenetic evidence (Ruhfel *et al.*, 2011). The three river-weed subfamilies can be distinguished by floral and capsule structures. There are three carpels (capsule valves) in the Tristichoideae and two carpels in Podostemoideae and Weddellinoideae. The Podostemoideae have their flowers enclosed by a sack-like or tubular cover (‘spathella’) that is lacking in *Weddellina*, the only genus in Weddellinoideae (Cook and Rutishauser, 2007; Koi and Kato, 2007; Kato, 2013). Molecular phylogenies strongly improved our knowledge of the evolutionary and biogeographical history of river-weeds, leading to many taxonomic changes and proposals for regrouping (Kita and Kato, 2001; Moline *et al.*, 2007; Thiv *et al.*, 2009; Ruhfel *et al.*, 2011; Tippery *et al.*, 2011; Koi *et al.*, 2012; Khanduri *et al.*, 2014). Promising studies published from the Japanese schools of Imaichi and Kato allow deeper insights into the developmental genetics of various Podostemaceae. Because the two main subfamilies, Tristichoideae and Podostemoideae, are morphologically divergent, we will present them in separate sections below.

### The morphological significance of vegetative bodies in Podostemaceae

The enormous degree of morphological variability makes comparative studies of Podostemaceae a challenging task. Several morphological peculiarities of the family do not fit into the classical architecture of angiosperms (Warming, 1891; Willis, 1902; Arber, 1920; Wardlaw, 1965; Mohan Ram and Sehgal, 1992; Rutishauser, 1997; Cook and Rutishauser, 2007). Various Podostemaceae have dorsiventrally flattened photosynthetic bodies that adhere to the hard substrate in rivers and waterfalls. The morphological significance of these flattened ribbons and crusts is still a subject of dispute. They have been interpreted as creeping roots (root crusts) or creeping stems (shoot crusts), depending on the species (subfamily) and the interpreting botanist. The conventional demarcation of the flattened photosynthetic body into root and shoot is often not obvious. Indian and French botanists in particular chose the neutral and descriptive term ‘thallus’ (without implying any homology to thalloid liverworts) because they doubted that the vegetative body of the Podostemaceae is homologous to the vegetative organs of conventional angiosperms with a CRS bauplan (Mohan Ram and Sehgal, 1992, 2001; Schnell, 1998; Sehgal *et al.*, 2002, 2007). As already described by Warming (e.g. 1891), Rutishauser (1997), Ota *et al*. (2001) and Koi *et al*. (2006), we adopt here – for convenience – the CRS model with its structural categories roots and shoots (including stems and leaves). Thus, we use the term ‘root’ in Podostemaceae for dorsiventrally flattened photosynthetic structures (ribbons, crusts) when endogenous shoot buds develop without exogenous leaves (Figs 1C–E, 2E, F, 3D, 7A–C and 8A, B). The root cap (calyptra) may be present or absent (Figs 2E, 6B and 8A). The term ‘stem’ (i.e. shoot axis) is applied to a photosynthetic body that develops exogenous leaves (Figs 2B, 3E, F, 4D, E and 6C). ‘Roots’ are usually fixed by adhesive hairs to the rocky substrate, whereas ‘shoots’ are fixed to the rock with a basal holdfast only. Opposite situations are found in two Asian genera: the podostemoid genus *Polypleurum* has ‘roots’ with free-floating parts up to 50 cm long, resembling *Fucus* kelp (Fig. 1B). The tristichoid genus *Dalzellia* has dorsiventrally flattened ‘shoots’ (with exogenous leaves on the upper surface) attached to the rock by adhesive hairs below (Fig. 4, see below for more details). Seedlings in Podostemaceae have two cotyledons (Fig. 3A). The radicle and plumule are usually short lived. The development continues by lateral outgrowths of the hypocotyledonary region, giving rise to adventitious (secondary) roots and shoots (Cook and Rutishauser, 2007; Koi and Kato, 2010; Katayama *et al*., 2011; Kato, 2013).

## THE RIVER-WEED PUZZLE. PART 1. PODOSTEMACEAE-TRISTICHOIDEAE: FROM A CLASSICAL ROOT–SHOOT (CRS) BAUPLAN TO DORSIVENTRALLY FLATTENED SHOOTS LACKING ROOTS

### Flowers in Podostemaceae – Tristichoideae with stable bauplans

The overall floral morphology is conserved in Tristichoideae. The flowers in this basal and rather small subfamily (six genera, approx. 18 species) have stable (developmentally robust) bauplans. All the tristichoid taxa share trimerous flowers with little variation in stamen number (Kato *et al.*, 2003; Kato, 2006, 2013; Khanduri *et al.*, 2014). The most complete flowers are found in *Dalzellia* and *Indotristicha*: a trimerous perianth (protecting the flower, fused to some degree), three stamens and a trimerous superior ovary with three locules (Figs 3B and 4F), maturing as many-seeded capsules. The flowers in the remaining tristichoid genera look similar, except for the reduction of stamen number from three to two and one in *Terniopsis* and *Tristicha*, respectively. The flowers in Tristichoideae are inserted on branched leafy stems, usually arising from exogenous buds. Cups (cupulae) of scaly leaves, which are fused into one vascularized collar-like unit around each flower**,** are a synapomorphy of the *Dalzellia–Indotristicha* sub-clade (Fig. 4B; Rutishauser and Huber, 1991; Koi *et al.*, 2012). The robustness in floral development of Tristichoideae is in contrast to their vegetative bodies which are highly plastic.

### *Tristicha* and *Terniopsis* as related genera with similar morphologies (Fig. 2)

*Tristicha* and *Terniopsis* (also *Indotristicha)* possess photosynthetic short-lived shootlets (called ‘ramuli’, singular ‘ramulus’). These ramuli are fine axes (up to a few centimetres long) that carry scale-like leaves along three rows (*Tristicha* and *Terniopsis*, Fig. 2A–F; Fujinami and Imaichi, 2009). Young ramuli show conical, slightly curved apical meristems (Fig. 2D). *Dalzellia* and *Indodalzellia*, however, lack ramuli (see next paragraphs). All tristichoid genera (except *Tristicha*) are restricted to Asia (Kato, 2006, 2013). *Tristicha* is the only genus that occurs with one polymorphic species (*T. trifaria*) in both the New World (America from Mexico South to Northern Argentina) and the Old World (Africa, Madagascar and the Mascarene Islands). Before molecular data were available, even populations (forms) from East Asia to North-Eastern Australia were added to *Tristicha* because they all share ‘ramuli’ with scale-like leaves in three lines (Fig. 2F). Due to molecular data showing paraphyly (e.g. Koi *et al.*, 2012), it became obvious that the *Tristicha*-like Asian to Australian taxa need to be separated as genera (*Cussetia*, *Terniopsis*). *Tristicha* differs from *Terniopsis*: usually one (rarely two) stamen per flower and a capless root in *Tristicha* (Fig. 2E), and usually two (rarely three) stamens per flower and a capped root in *Terniopsis*. In the polymorphic *Tristicha trifaria sensu lato* (*s.l.*), some African populations (accepted as *T. alternifolia* until 1950, e.g. in Angola) show elongated and branched tristichous ramuli whereas other African and all New World populations [accepted as *T. hypnoides*, syn. *T. trifaria sensu stricto* (*s.s.*) in earlier days] have rather short ramuli with dense rows of scale-leaves (Fig. 2A–C). Fujinami *et al.* (2013) studied the developmental morphology of *T. trifaria s.l.*, showing the complex formation of a basal shoot disk that is closely attached to the substrate. Fujinami *et al.* (2013) accepted for the basal disks of *Tristicha* congenital fusion of various shoot axes orders, as will be discussed below under *Dalzellia*.

### *Dalzellia–Indotristicha* lineage: saltational loss of root–shoot bauplan in *Dalzellia* with a crustose vegetative shoot, as compared with the closely related *Indotristicha* with roots and shoots (Figs 3 and 4)

*Dalzellia* and *Indotristicha* are sister genera in Asian Tristichoideae with distinctly different morphologies. The best known example is represented by the two species *Dalzellia zeylanica* and *Indotristicha ramosissima* from South Asia (especially South India and Sri Lanka). Both *Dalzellia* and *Indotristicha* are closely related genera forming a sub-clade: the monotypic genus *Indotristicha* is sister to the genus *Dalzellia* that contains five species (Koi *et al.*, 2012; Kato, 2013).

*Indotristicha ramosissima* appears to be a rather conventional flowering plant with regard to its vegetative bauplan. Like *Tristicha* and allies (see above), *Indotristicha* has short-lived photosynthetic shootlets (‘ramuli’). Unlike *Tristicha*, the scale-like leaves in the *I.* ramuli are inserted spirally or irregularly (Fig. 3C, E, F). The plant body of *I. ramosissima* consists of ribbon-like adhesive roots (with cap) and root-borne, branched shoots up to 64 cm long (Rutishauser and Huber, 1991). Seedlings of *I. ramosissima* have secondary roots arising exogenously from the hypocotyl (Fig. 3A). The radicle stops growth after the formation of some adhesive hairs. The plumule usually is short-lived. The secondary roots elongate and branch before giving rise to root-borne shoots. They arise from endogenous buds together with flattened holdfasts that fix the outgrowing leafy stems to the rock (Fig. 3D).

*Dalzellia zeylanica s.l.* (including closely related species such as *D. ubonensis*) deviates strongly from the conventional bauplan of flowering plants. The shoot is crustose (foliose) and adheres to the rocky substrate like a foliose lichen, whereas the root is lacking (Fig. 4A, B, D, E; Imaichi *et al.*, 2004; Fujinami and Imaichi, 2015). The crustose shoot shows dorsiventral construction: scaly leaves are restricted to its margin (‘marginal leaves’) and its upper surface (‘dorsal leaves’). No leaves are found on the lower surface that is attached to the substrate. The crustose shoot of *D. zeylanica* was explained as a result of congenital fusion of various shoot axes orders (‘coenosome’ *sensu* Jaeger-Zuern, 1992, 1995, 1997, 2003). This interpretation was taken over by Imaichi *et al.* (2004), Fujinami *et al.* (2013) and Fujinami and Imaichi (2015). According to Jaeger-Zuern (2003), the shoot apical meristem (shorter: shoot meristem) of *D. zeylanica* is not conical (in contrast to *T. trifaria* and *I. ramosissima*), but wide and flat. The crustose shoot of *D. ubonensis* (*D. zeylanica s.l.*) appears to be formed by zonal growth in the common region behind several shoot meristems, as well as by marginal meristems that spread among the shoot meristems (Fujinami and Imaichi, 2015). Flowers in *D. zeylanica* arise from endogenous buds in the cortex of the dorsiventrally flattened shoots. When the crustose shoots start to emerge at the end of the monsoon, most of the exogenous scale-like leaves are dropped (erased). Then there is meristematic activity inside the shoot cortex below the upper surface, giving rise to rosettes of scale-like leaves and finally to flower buds**,** each of which is surrounded by a fringed cup (‘cupule’) (Fig. 4B, C).

### *Indodalzellia gracilis* (Fig. 5) as the missing link between *Dalzellia* and *Indotristicha*?

*Indodalzellia gracilis* was discovered and described as a new taxon by Mathew *et al.* (2001). This species, endemic to South India (Kerala), was first thought to be a member of *Dalzellia* (as *D. gracilis*). In agreement with molecular findings, it was put into a genus on its own (Koi *et al.*, 2009). Phylogenetically it is sister to the sub-clade consisting of *Indotristicha* and *Dalzellia*. *Indodalzellia* is derived from the paraphyletic *Tristicha* and *Terniopsis* but sister to the *Dalzellia–Indotristicha* lineage (Koi *et al.*, 2012). With respect to its morphological features, *I. gracilis* can be regarded as intermediate between *Indotristicha* and *Dalzellia*. *Indodalzellia* (Fig. 5A) has ribbon-like creeping roots, being convex on the upper side and slightly concave or planar below, similar to *Indotristicha*, *Tristicha* and *Terniopsis* (Figs 2E, F and 3D). The *Indodalzellia* root is capless like the *Tristicha* root. When the roots cannot directly fix to the rocky substrate, finger-like holdfasts grow out along the root flanks turning downwards until they reach the substrate (Fig. 5C). They stick to the rock by adhesive hairs (Fig. 5D). The root-borne shoots of *Indodalzellia* arise from the root flanks (probably from endogenous buds, as indicated by Koi *et al.*, 2009). Finally, the strongly flattened shoots (stems) are fixed to the substrate on the lower side. They carry dimorphic scale-like leaves on their upper surface, larger ones along the margin and smaller ones on the dorsal surface (Fig. 5E). This pattern with two kinds of scales is identical to what is known from *D. zeylanica s.l.* (Fig. 4).

### Morphology and developmental genetics of the dorsiventrally flattened shoots in *Dalzellia* and *Indodalzellia*

Fujinami and Imaichi (2015) studied the developmental morphology (including genetics) in *D. ubonensis* (from Thailand) that is nested in *D. zeylanica s.l.* (Koi *et al.*, 2012). Thus, what Fujinami and Imaichi found in *D. ubonensis* is also valid to some degree for *D. zeylanica s.s.* (from South India and Sri Lanka). A set of unique shoot meristems is active as a meristematic line (crest) along the margin of the dorsiventrally flattened shoot. Fujinami and Imaichi (2015) found expression of the *KNOX* gene (*DuSTM*) and the *WOX* gene (*DuWUS*) along growing margins of crustose shoots in *D. ubonensis. Dalzellia* **(**and to a minor degree also *Tristicha* and *Indodalzellia*) may show a genetically fixed type of shoot fasciation (as discussed by Fujinami and Imaichi, 2015). Such a view coincides with the shape changes of shoot apical meristems (SAMs) in *Arabidopsis thaliana*, which are due to changes in the specific gene networks including the interaction of *WUSCHEL* (*WUS*) and *CLAVATA* (*CLV*) **(**Fujita and Kawaguchi, 2011). Due to fasciation, ordinary SAMs change from a conical shape (radial symmetry) to a meristematic line (crest) along which new leaves and lateral shoots are initiated. The switch from an *Indotristicha*-like growth form to the flattened shoot crusts in *Dalzellia* (and *Indodalzellia*) perhaps happened within a short time, as hypothesized already by Imaichi *et al*. (2004): ‘The saltational evolution of the *Dalzellia zeylanica* bauplan may be due to drastic early ontogenetic changes, such as the appearance of secondary shoots in the epicotylar region and loss of the root, as well to modifications, such as flattening and adherence of the shoot compensating functionally for loss of the root.’ Kato (2013, p. 166) repeated: ‘It is likely that saltational evolution happened in this lineage.’

## THE RIVER-WEED PUZZLE. PART 2. PODOSTEMACEAE-PODOSTEMOIDEAE: FROM A NEARLY CLASSICAL ROOT–SHOOT (CRS) BAUPLAN TO DORSIVENTRALLY FLATTENED ROOTS, AND SHOOTS THAT LACK OBVIOUS APICAL MERISTEMS

### Spathella as a synapomorphy of Podostemoideae

The Podostemoideae (comprising 47 genera and approx. 290 species) is the largest of the three river-weed subfamilies. It is distinguishable from the Tristichoideae and Weddellinoideae by the presence of a thin non-vascularized tubular cover, the spathella, which encloses the young flower (Fig. 7D). The monotypic genus *Diamantina* (being endemic to Minas Gerais, Brazil) is the only podostemoid member known having some flowers without tubular spathellas (Fig. 6D, E; Rutishauser *et al.*, 2005). *Diamantina* appears to be sister to the remaining Podostemoideae in molecular phylogenies (Koi *et al.*, 2012). Thus, open subtending bracts (replacing tubular spathellas) in *Diamantina* may be viewed as a plesiomorphic condition (or atavism) whereas tubular spathellas appear to be a synapomorphy of Podostemoideae.

### Podostemoideae with rather stable floral bauplans: from polyandrous flowers in the New World to oligostemonous flowers elsewhere

Podostemoid flowers show some variation with respect to the number of stamens and tepals, the latter being inconspicuous throughout. Various American podostemoids have insect-pollinated (and even scented) flowers with a whorl of 6–12 showy (white to pink) stamens and as many inconspicuous tepals (as observable in several species of *Apinagia* or *Marathrum*, Fig. 1A). Other entomophilous podostemoids increase the number of showy stamens per flower up to 40 (e.g. various species of *Apinagia*, *Marathrum*, *Mourera* and *Rhyncholacis*; see Tavares, 1997; Cook and Rutishauser, 2007, Kato, 2013).

In many New World Podostemoideae (e.g. *Podostemum*) and all Old World genera (mainly Africa and Asia) the flowers become dorsiventral by stamen loss on one side, finally leading to flowers with one stamen (e.g. Fig. 8C) or two stamens. All podostemoids having only 1–2(–4) stamens per flower depend on wind pollination (i.e. flowers less conspicuous, without scent). If two stamens per flower are present, their filaments usually have a common base (‘andropodium’), leading to a Y-shaped structure with two anthers (e.g. Figs 1B and 7D). The family name ‘Podostemaceae’ (based on the genus *Podostemum*) refers to this distinctive feature. Perhaps as an adaptation to anemogamy, various African podostemoids (but none outside Africa) evolved firm (i.e. non-decaying) pollen dyads instead of having single pollen grains (e.g. *Ledermanniella linearifolia*, Fig. 7E; Ameka *et al.*, 2003; Grob *et al.*, 2007*b*; Moline *et al.*, 2007).

All podostemoid flowers have superior ovaries with two fused carpels forming two locules, or only one due to septum loss, as typical for several derived African genera (e.g. Fig. 7F: *Ledermanniella*; Ameka *et al.* 2003). In all non-African groups, the flower buds are upright and sessile (Fig. 6D, E), whereas in most African Podostemoideae the flower buds are completely inverted (Figs 7D and 8C). An inverted floral bud inside the spathella may be seen as an adaptation that increases the speed of spathella rupture and the quick onset of anthesis once the plants emerge from the water (Cook and Rutishauser, 2007).

### Continuum from ribbon-like to crustose roots

Root ribbons (width up to 10 mm) with endogenous shoot formation along the root margins are found in many Podostemoideae, e.g. *Stonesia ghoguei* (Fig. 8A). The ribbon-like roots of several podostemoids may have tips covered by a dorsiventral root cap (Fig. 6B). Capped roots in Podostemoideae usually show the endogenous origin of lateral roots. In other podostemoids with ribbon-like roots the tips lack caps, and root branching happens exogenously (Fig. 8A, B). Various Old World podostemoids possess even broader roots (up to several centimetres wide) with endogenous shoots arising from the upper surface (e.g. Figs 1C and 7A, B). These disk-like roots in Podostemoideae are usually labelled as ‘crustose’ or ‘thalloid’, also ‘foliose’ because they resemble foliose lichens (Ota *et al.*, 2001; Hiyama *et al.*, 2002; Kato, 2004). Crustose roots in Africa and Asia may have evolved three or four times independently from groups having ribbon-like roots (Koi *et al*., 2006; Moline *et al.*, 2007). Possible root transformation series in Podostemoideae were illustrated in Rutishauser and Moline (2005, their fig. 6).

### Prominent shoots with non-axillary branching and terminal double-sheathed leaves

In most angiosperms, axillary branching along stems entails the production of a lateral shoot bud in the distal axil of a subtending leaf. Conventional axillary branching as known from typical angiosperms occurs only rarely in Podostemoideae, e.g. in *Saxicolella submersa* (Ameka *et al.*, 2002). In many Podostemoideae (e.g. *Ledermanniella bowlingii* and *Podostemum ceratophyllum*) there are leaves with two sheaths that are inserted laterally and opposite each other. Such leaves have been called double-sheathed or ‘dithecous’ by Warming (e.g. 1891) and others (Rutishauser, 1997; Rutishauser and Grubert, 2000; Ameka *et al.*, 2003; Rutishauser *et al.*, 2003; Moline *et al.*, 2006; Ghogue *et al.*, 2009). The occurrence of double-sheathed leaves among conventional (i.e. single-sheathed) leaves allows the stem to branch by a peculiar process that, due to the absence of a more appropriate term, may be called ‘bifurcation’. As long as a stem is developing single-sheathed leaves, it grows in a monopodial manner. Then, a double-sheathed leaf appears in the terminal position, giving rise to new shoot modules (daughter shoots) in each sheath, or one of the two sheaths is replaced by a flower instead of a daughter shoot. The many-flowered sword-like inflorescences (up to 60 cm long, including the stalk) of *Mourera fluviatilis* from northern South America consist of two rows of double-sheathed bracts that are initiated in a basipetal order (Rutishauser and Grubert, 1999). The double-sheathed leaves in podostemoids resemble to some degree laterally flattened (i.e. ensiform) leaves in more typical angiosperms such as *Acorus* and *Iris* (Jäger-Zürn, 2000, 2003, 2007).

### Floral sites along roots, shoots and leaves in Podostemoideae

Unlike conventional flowering plants, floral buds in several Podostemoideae are initiated nearly everywhere on the vegetative body. Flowers arise from endogenous buds on the upper surface of flattened roots (e.g. in *Ledermanniella linearifolia*, Fig. 7A, B), or they are initiated along the stems (e.g. *Stonesia ghoguei*, Fig. 8D), starting as endogenous buds by dedifferentiation of parenchyma cells inside the stem cortex. The buds finally protrude the stem periphery, rupturing the outer cortical layers and epidermis (Pfeifer *et al.*, 2009). Endogenous formation inside the undamaged stem protects the flower buds from the rushing and damaging water. They protrude and open quickly when the water level has dropped sufficiently. A few African Podostemoideae show epiphyllous flowers that are inserted on leaves. In *S. ghoguei* they are initiated in the clefts (angles) of forked leaves (Fig. 8E, F; Moline *et al.*, 2007; Pfeifer *et al*., 2009). Both epiphyllous flowers and endogenous bud formation inside the stem may be understood as the result of ectopic expression of flower identity (Rutishauser and Moline, 2005; Rutishauser *et al.*, 2008; Tsukaya, 2014).

### Shoot apical meristems cryptic or even lacking in Podostemoideae

Tristichoideae and Weddellinoideae usually have obvious SAMs that produce laminar leaves on their flank (Figs 2D and 3F; for *Weddellina,* see Koi and Kato, 2007). On the other hand, in the more derived subfamily Podostemoideae, the shoots lack recognizable SAMs with permanent stem cells. They are cryptic (indistinct) and difficult to observe in the vegetative shoots of Podostemoideae (Rutishauser *et al.*, 2003; Jäger-Zürn, 2007). According to Japanese studies (e.g. Imaichi *et al.*, 2005; Katayama *et al.*, 2008, 2010, 2013; Koi and Kato, 2010), the leaf primordium in vegetative shoots develops from the base of the opposing second youngest leaf primordium. The initiation of a new leaf primordium appears to be associated with degeneration of neighbouring cells, as shown by Imaichi *et al.* (2005), Koi *et al.* (2005), and Koi and Kato (2010) for Asian Podostemoideae. Such leaf formation is repeated, resulting in a chain of leaves.

### Developmental genes involved in bauplan deviations of Podostemoideae as compared with Tristichoideae

Katayama *et al.* (2010, 2013) investigated the mechanisms underlying shoot development in Podostemaceae by expression analysis of key developmental regulatory gene orthologues in model eudicots. *STM* (*SHOOT MERISTEMLESS*) and *WUS* are necessary for the formation and maintenance of the SAM in eudicots (including arabidopsis), and *ARP* (*ASYMMETRIC LEAVES1/ROUGH SHEATH2/PHANTASTICA*) promotes leaf identity (Gallois *et al.*, 2002; Langdale and Harrison, 2008; Takeda and Aida, 2011). In the tristichoid shoots (e.g. *Terniopsis minor*), *STM* and *WUS* orthologues were expressed in the prominent SAMs, as in model plants (e.g. arabidopsis). In the podostemoid shoots (e.g. *Hydrobryum japonicum*, *Cladopus doianus* and *Zeylanidium lichenoides*) with cryptic (indistinct) SAMs, the *STM* and *WUS* orthologues are expressed in the initiating leaf primordia. The leaves in Podostemoideae produce cryptic meristems near their bases, which bulge as a new SAM and subsequently differentiate into a terminal leaf. *WUS* expression soon disappears in the developing leaf primordia, and *STM* expression is restricted to their basal parts, whereas *ARP* is expressed in their distal parts in a complementary pattern to *STM* expression. Thus, the SAM in podostemoids (at least Asian ones) appears to have been converted into a single, terminal leaf by losing the expression of genes (*STM/WUS*) responsible for continued stem growth and gaining expression of genes (*ARP*) that promote leaf identity. As a result, the leaves have ‘a mixture of shoot and leaf, showing fuzzy morphology’ (Kato, 2013, p. 45). According to the evolutionary model proposed by Katayama *et al.* (2010, 2011, 2013), the shoots in Podostemoideae grow by repetitive formation of stem–leaf mixed organs and this pattern is derived from the sympodial shoot branching of Tristichoideae and Weddellinoideae. The early loss of embryonic shoot and root meristems (i.e. short-lived plumules and radicles) in river-weed seedlings is similar to *MONOPTEROS* and other mutants known in arabidopsis (Treml *et al.*, 2005; Katayama *et al.*, 2011; Takeda and Aida, 2011).

## THE BLADDERWORT PUZZLE: THE EVOLUTION OF UNUSUAL MORPHOLOGIES IN THE LENTIBULARIACEAE (Figs 9–14)

### Carnivory and molecular systematics

The Lentibulariaceae are carnivorous plants that usually grow in nutrient-poor habitats. There are flypaper traps (*Pinguicula*), eel traps = lobsterpot traps (*Genlisea*), and bladder traps that conduct suction in <1 ms (*Utricularia*) (Adamec, 2011; Vincent *et al.*, 2011). Unlike *Genlisea* and *Utricularia*, the genus *Pinguicula* (butterworts) is characterized by roots, stem and leaves. The *Pinguicula* roots are usually weak and short-lived, even lacking a root cap in some species (Rutishauser and Isler, 2001; Adlassnig *et al.*, 2005; Kirchoff *et al*., 2008). The leaves of *Pinguicula* are entire and form a basal rosette. *Genlisea* and *Utricularia* are morphologically more divergent than *Pinguicula*. *Utricularia* is closely related to *Genlisea*, with *Pinguicula* being sister to a *Genlisea–Utricularia* sub-clade. This view is supported by floral morphology as well as molecular data. The radiation in Lentibulariaceae (including basal Utricularias) started with the terrestrial habit, and both aquatic and epiphytic species in *Utricularia* represent derived conditions (Jobson *et al*., 2003; Müller *et al*., 2004, 2006; Müller and Borsch, 2005; Guisande *et al*., 2007; Schäferhoff *et al*., 2010; Veleba *et al*., 2014). As part of this pictorial report, we place emphasis on aquatic bladderworts. Approximately 50 *Utricularia* species are aquatic (or amphibious), growing in standing, nutrient-poor humic waters. About 34 of these aquatic species belong to the section *Utricularia* within *Utricularia* subgenus *Utricularia* (according to Taylor, 1989), which is identical (or nearly so) to a sub-clade in molecular analyses.

### Flowers in Lentibulariaceae with stable bauplans (Fig. 11F)

The three genera (*Genlisea*, *Pinguicula* and *Utricularia*) in this family have flowers with a stable (developmentally robust) bauplan (Lloyd, 1942; Degtjareva and Sokoloff, 2012). As typical for several families of the Lamiales (Asteridae) within eudicots, the zygomorphic insect-pollinated flowers consist of a bilabiate sympetalous corolla, made up of five connate petals with a spur usually containing nectar (Hobbhahn *et al*., 2006; Fleischmann, 2012*a*; Clivati *et al*., 2014). The resulting flower type is called a masked flower (snap-dragon type blossom) because the entrance to the throat and nectar spur is sealed to some degree. In all Lentibulariaceae, the androecium consists of two stamens that are hidden inside the upper corolla lip. The gynoecium consists of a superior ovary topped by a two-lobed stigma (being sensitive in various Utricularias). Its arrangement relative to the androecium usually prevents autogamy (self-pollination) although some Utricularias are known to be self-compatible or even cleistogamous inbreeders (Jérémie, 1989; Khosla *et al*., 1998; Clivati *et al*., 2014). The main difference in Lentibulariaceae is found in sepal number: *Pinguicula* and *Genlisea* with five calyx lobes per flower, *Utricularia* subgenus *Polypompholyx* with four calyx lobes and all remaining bladderworts with two sepals per flower (Fig. 11F; Grob *et al*., 2007*a*; Degtjareva and Sokoloff, 2012; Fleischmann, 2012*a*, his fig. 103). If there is more than one flower per raceme (as typical for *Genlisea* and *Utricularia*), the lateral ones are subtended by a bract (Fig. 11A, F). Along the inflorescence axes all Utricularias behave like typical angiosperms, showing axillary branching. Therefore, bladderworts can be viewed as one phase only misfits (cf. Minelli, 2015*b*) because they return to the conventional branching pattern while forming flowers (Rutishauser and Isler, 2001).

### Released bauplans in the vegetative (non-flowering) parts of *Utricularia* and (less so) in *Genlisea*

Kaplan (1998, Vol. 3, p. 75) wrote on the unusual morphologies in *Utricularia*: ‘While its flowers and inflorescences are fairly stereotypical, its species exhibit an incredible polymorphism vegetatively, which superficially, at least, seems to defy all the principles of vascular plant organography and have caused no end of interpretive problems and arguments.’ Axillary shoot branching as typical for conventional seed plants (with daughter modules arising from the distal axils of subtending leaves) is still found in leaf rosettes of *Pinguicula* (Grob *et al*., 2007*a*). Axillary branching, however, is lacking or less obvious during vegetative growth of *Genlisea* and *Utricularia* (Lloyd, 1942). *Genlisea* (usually regarded as rootless) and *Pinguicula* (still with roots) can be viewed as slight modifications of the CRS model, whereas strongly released (decanalized) body plans are typical in the vegetative parts of all bladderworts (Jobson and Albert, 2002; Jobson *et al*., 2004). Lloyd (1942, p. 213) was aware of this fact while writing on *Utricularia* in general: ‘They represent a complex and puzzling morphology. They are entirely rootless, even in the embryonic condition. The distinction between stem and leaf is vague. Only in the inflorescence and in certain shoots (air-shoots of *U. vulgaris* etc.) is the morphology easily recognizable.’ Within the Lentibulariaceae, the loss of the CRS bauplan in bladderworts was not correlated to a switch from terrestrial to aquatic habitats because the released bauplans occurred already in basal Utricularias (including subgenus *Polypompholyx*) that are terrestrial taxa although their bladders need to be water-filled for firing and catching prey (Taylor, 1989; Reut and Fineran, 2000).

### Vagueness (fuzziness) of organ identities in Utricularia (bladderworts): ‘stolons’ and ‘leaves’ as neutral terms for describing the vegetative bodies in the *Genlisea–Utricularia* lineage

Taylor (1989, p. 6) wrote in the introduction to his *Utricularia* monograph: ‘For taxonomic and descriptive purposes, whatever their true or theoretical nature, it is desirable to have a consistent terminology for the various organs.’ Most Utricularias produce root-like organs or runners that were called ‘stolons’ (horizontal shoots) and ‘rhizoids’ (anchoring organs) by Taylor (1989) and Adlassnig *et al.* (2005). They are labelled as ‘runner stolons’ and ‘anchor stolons’, respectively, by Reut and Fineran (2000). In addition there is a range of leaf-like organs (called ‘leaves’ by Taylor 1989). The vagueness (fuzziness) of organ identities in *Utricularia* allowed contradictory interpretations, as already discussed by Arber (1920). Stolons and rhizoids have been viewed as stem homologues, including phyllomorphic shoots (Troll and Dietz, 1954; Fleischmann, 2012*a*), as leaf homologues (Goebel, 1891; Kumazawa, 1967; Kaplan, 1998) or even as ‘fuzzy organs’ blending (amalgamating) the developmental programmes of leaves and shoots (Rutishauser and Sattler, 1989; Sattler and Rutishauser, 1990; Rutishauser, 1999; Rutishauser and Isler, 2001). Thus, it is still a question of biophilosophical outlook if botanists choose a classical or a fuzzy perspective for describing and interpreting the vegetative bodies in bladderworts (although the fuzzy view accords more with what is observable). In order to obtain an impression of the vast morphogenetic possibilities found in the *Genlisea–Utricularia* lineage, the developmental morphology of some *Utricularia* members (Figs 9–13) and one *Genlisea* species (Fig. 14) will be presented below.

### Branching patterns and structural units as observable in the vegetative bodies of aquatic bladderworts (subgenus *Utricularia* – section *Utricularia*, see branching schemes Figs 9 and 10G)

The developmental morphology of aquatic bladderworts (section *Utricularia*) such as *Utricularia aurea*, *U. australis*, *U. foliosa*, *U. gibba*, *U. macrorhiza*, *U. stellaris* and *U. vulgaris* (Figs 9–11) is quite well known (Arber, 1920; Lloyd, 1942; Troll and Dietz, 1954; Rutishauser and Sattler, 1989; Sattler and Rutishauser, 1990; Rutishauser, 1993; Chormansky and Richards, 2012). I give here a short overview of the branching patterns of aquatic bladderworts because both *Utricularia* species with published genome (transcriptome) analyses belong to this group: *U. gibba* and *U. vulgaris* (Ibarra-Laclette *et al*., 2011, 2013; Veleba *et al*., 2014; Bárta *et al*., 2015; Carretero-Paulet *et al*., 2015*a*, b). Each ‘leaf’ or leaf-like organ in the aquatic bladderworts (sect. *Utricularia*) consists of two branched lobes that can be equal in size, both carrying several bladders, as observable in *U. australis* (Fig. 10A). Alternatively, the two ‘leaf’ lobes are different in size and trap number, with the upper lobe short, photosynthetic and provided with few bladders, whereas the lower lobe lacks chlorophyll, elongates and turns downwards into deeper water and mud, and is provided with many bladders (as found in *U. foliosa*; Sattler and Rutishauser, 1990). The bladders (traps) of aquatic bladderworts (sect. *Utricularia*) carry two branched dorsal appendages near the mouth, besides a few additional bristles (Fig. 10B). Growing stolon tips are coiled upwards, showing circinate vernation, with bifid leaf primordia inserted in a distichous phyllotaxis pattern along the two lateral sectors (stolon flanks, Fig. 10C). The growing tips of young ‘leaf’ lobes resemble the stolon tips, although they are less coiled (Fig. 10D). Thus, the stolons (also called ‘watershoots’) and the two-lobed ‘leaves’ have similar developmental pathways, indicating leaf–shoot indistinction (Sattler and Rutishauser, 1990; Rutishauser *et al*., 2008). The dorsiventral stolon symmetry is obvious with respect to the positional arrangement of inflorescence buds and (in some but not all aquatic Utricularias) so-called ‘air-shoots’ which are tiny filamentous stolons (with scale-like leaves) reaching the water surface. Both inflorescence buds and air-shoots arise from the dorsal (upper) sector of the main stolon in aquatic Utricularias (e.g. *U. australis*, *U. aurea* and *U. stellaris*, Figs 10D–G and 11D). The main stolons give rise to daughter stolons (branch watershoots), usually from near the inflorescence base (Fig. 10D, G). Several aquatic Utricularias (e.g. *U. australis*, *U. aurea* and *U. gibba*) show additional stolon-like or root-like appendages arising from the lower end of the peduncle (inflorescence stalk), without being subtended by bracts or leaves. They were labelled as ‘anchor stolons’ or ‘rhizoids’, because they serve as anchoring organs in order to keep the inflorescence upright (Arber, 1920; Lloyd, 1942; Taylor, 1989). In a few aquatic species such as *U. stellaris*, the anchor stolons (rhizoids) at the peduncle base are replaced by a whorl of spongy floats (inflated buoys, Fig. 11B, C), again helping to keep the inflorescence in an upright position during anthesis (Lloyd, 1942; Khosla *et al*., 1998). In aquatic species such as *U. aurea*, some populations produce floats whereas others have anchor stolons (Rutishauser, 1993). Various aquatic Utricularias living in cold-temperate climates (e.g. *U. australis*, *U. macrorhiza* and *U. vulgaris*) are perennial by surviving with turions **(**winter-buds) at the bottom of ponds and lakes (Taylor, 1989; Guisande *et al*., 2007; Adamec, 2010; Plachno *et al*., 2014*b*). Some of these are vegetative apomicts (e.g. *U. australis* and *U. bremii*) producing flowers but no seeds.

The developmental architecture of *Utricularia gibba* (also belonging to the aquatic bladderworts of section *Utricularia*) was illustrated by Chormanski and Richards (2012, their fig. 19). Their ‘architectural model’ for *U. gibba* needs improvement. Chormanski and Richards (2012) described the ‘leaves’ (leaf-like structures) in *U. gibba* as arranged spirally along the stolon; and they accepted daughter stolons (secondary stolons) and inflorescences as axillary outgrowths subtended by ‘leaves’ (leaf-like structures). According to Lloyd (1942) and Rutishauser (unpubl. data), *U. gibba* shows a distichous arrangement of the ‘leaves’, inserted along both flanks (lateral sectors) of the stolons. They show dorsiventral symmetry, with secondary stolons (lateral branches) and inflorescences arising from near the upper edge of the leaf insertion, but not in the leaf axil (Figs 9 and 10G).

### Terrestrial Utricularias, e.g. *Utricularia sandersonii*, showing runner stolons with a dorsal row of ‘leaves’ (Fig. 12)

*Utricularia sandersonii* **(**from South Africa) belongs (together with *U. livida* and approximately another nine species) to the mainly African section *Calpidisca* within *Utricularia* subgenus *Bivalvaria* (Taylor, 1989; Veleba *et al*., 2014). They all are small terrestrial annuals, with capillary runner stolons (approx. 0·2 mm thick), with petiolate entire leaves (total length up to 15 mm, including obovate lamina in *U. sandersonii*, Fig. 12A, B). Most of the bladder traps are inserted along the capillary stolons (Fig. 12D) or arise from the midrib and petiole on the lower leaf sides (Brugger and Rutishauser, 1989; Rutishauser and Isler, 2001, their fig. 15). The traps have their mouth fringed with radiating rows of gland-tipped hairs (Fig. 12E). As usual for all *Utricularia* traps, there are mainly four-armed glands (so-called quadrifids) covering the inner bladder wall (Fig. 12F). The branching scheme of the stolons (Fig. 9) illustrates the situation found in *U. sandersonii* and other *Calpidisca* members (Brugger and Rutishauser, 1989): the stolon tips are straight (i.e. not coiled as in aquatic members of sect. *Utricularia*, Figs 10 and 11). The stolons nevertheless show a dorsiventral symmetry with respect to their morphogenetic potential of producing appendages: all leaves are inserted (‘riding’) along the upper (dorsal) sector (Figs 9 and 12A, B) whereas traps are inserted along the lateral sectors only (Fig. 12D). All additional outgrowths (such as inflorescences and daughter stolons) originate from buds along the upper stolon sector. The ‘leaves’ and their ‘axillary buds’ (rosettes) seem to be twisted 180° when compared with the axillary branching of conventional seed plants. The subtending leaf is in a more distal position along the stolon, whereas its axillary bud originates in a more proximal position (Fig. 12C). This inverse axillary (‘wrong’) position of rosettes along dorsal stolon sectors is also known from other non-aquatic Utricularias, e.g. *U. dichotoma* of subgenus *Polypompholyx*, and *U. longifolia* of subgenus *Utricularia* (Reut and Fineran, 2000; Rutishauser and Isler, 2001; see next paragraph).

### Epilithic to epiphytic Utricularias, e.g. *U. longifolia* and *U. alpina* (Fig. 9)

Unlike the tiny *U. sandersonii*, there are (mainly in tropical America) epilithic to epiphytic species that are much larger with respect to flower size (up to 6 cm) as well as size of vegetative parts such as leaves (up to 30 cm long) and stolons (tubers in *U. alpina* with diameter >1 cm). They belong to two sections within *Utricularia* subgenus *Utricularia.* Most epiphytic species (including *U. alpina*, *U. humboldtii* and *U. reniformis*) are members of section *Orchidioides* because their flowers resemble showy orchids (Jérémie, 1989; Taylor, 1989; Clivati *et al*., 2014). *Utricularia longifolia* belongs to sect. *Foliosa s.l.* (including *Psyllosperma*) as sister of sect. *Orchidioides* (Müller and Borsch, 2005; Veleba *et al*., 2014). Branching analyses of the vegetative bodies of these rather large plants were published by Troll and Dietz (1954), Brugger and Rutishauser (1989) and Rutishauser and Isler (2001). Members of sect. *Orchidioides* (e.g. *Utricularia alpina*) have coiled stolon tips (Fig. 9) whereas they are straight in *U. longifolia*. With respect to stolon branching and leaf position, all studied epiphytic (epilithic) Utricularias clearly exhibit stolon dorsiventrality, without outgrowths along the lower (ventral) sector and only tiny appendages (such as stalked bladders) along the lateral stolon sectors. *Utricularia longifolia* behaves similarly to *U. sandersonii* (Fig. 12C) with respect to the positions of leaves and axillary buds along the upper (dorsal) stolon sector, again with inverse position of the axillary bud and subtending leaf (Rutishauser and Isler, 2001, their figs 17 and 18). In *U. alpina*, both leaves as well as daughter stolons and inflorescences originate from extra-axillary meristematic buds (rosettes) along the upper (dorsal) stolon sector, not being associated with subtending leaves. The primordia arising from these meristematic buds show a delay with respect to their developmental fate. Thus, in early stages, it is not possible to decide if a primordium grows into a daughter stolon or into a foliage leaf (Brugger and Rutishauser, 1989; Rutishauser and Isler, 2001).

### Haptophytic Utricularias, e.g. *Utricularia neottioides*, coming close to the habit of Podostemaceae (Fig. 13)

There are a few bladderworts adapted to river habitats as Podostemaceae, growing as affixed perennials (haptophytes) in swiftly flowing water, with their feet attached to submerged rocks. Taylor (1989) added these rheophytic species to his sections *Avesicaria*, *Avesicarioides* and *Mirabiles*, which do not form a clade in molecular phylogenies (Müller and Borsch, 2005; Guisande *et al.*, 2007). Thus, the haptophytic habit evolved more than once in the genus *Utricularia*. As illustrated by *U. neottioides* (section *Avesicaria*; Fig. 13A–C), rheophytic Utricularias produce claw-like anchor stolons (rhizoids) that are provided with adhesive hairs (trichomes) along their lower (ventral) side. This is a nice example of convergence to the holdfasts in Podostemaceae, Hydrostachyaceae (eudicots) and seagrasses (monocots) such as *Posidonia* and *Phyllospadix* (Lloyd, 1942; van Steenis, 1981; Jäger-Zürn, 1998; Schäferhoff *et al*., 2010; Badalamenti *et al*., 2015).

### *Genlisea*, the corkscrew plants, as sister genus to bladderworts (Fig. 14)

With only 29 species *Genlisea* is the smallest of the three lentibulariaceous genera, occurring in tropical America, Africa and Madagascar. Because *Genlisea* and *Utricularia* are sister genera (Veleba *et al*., 2014), a short introduction to the vegetative *Genlisea* body will be given here, using *Genlisea repens* (from Brazil and adjacent countries) as an example. It grows with often submerged rosettes and stolons in shallow acidic water (Fleischmann, 2012*a*). Its vegetative body consists of spathulate green leaves (up to 4 cm long) and Y-shaped eel traps (up to 7 cm long) that function in wet soil (Fig. 14A, E, F). Because these traps resemble roots (at least to some degree) they were labelled as ‘rhizophylls’ (i.e. root-leaves) by Goebel (1891). They attract and trap soil protozoa as well as invertebrates and even algae (Plachno *et al*., 2007, 2008). Both green leaves and eel traps in *Genlisea* arise as exogenous primordia from the same SAM (Fig. 14B). As typical for heterophyllous plants, some primordia turn into green leaves above the water level or mud (Fig. 14D), whereas others give rise to one eel trap each (Fig. 14C). An early developmental stage of a *Genlisea* trap consists of a stalk with a distal mouth-like cavity and two embryonal bulges (Fig. 14C). These bulges will elongate and twist, leading to the two catching arms (‘corkscrews’) with a longitudinal slit each (Fig. 14F, G). In the meantime, the lower tube (including the digestion bulb) is formed (Fig. 14E).

### Trap evolution in Lentibulariaceae

Plachno *et al.* (2007) found similarities in the digestive hairs and their fine structural features of the traps in *Pinguicula*, *Genlisea* and *Utricularia*. Nevertheless, it is difficult to imagine evolutionary transitions between the immobile traps of *Pinguicula* and *Genlisea* (Fig. 14E, F) and the active traps in *Utricularia* (Figs 10B and 12D; Fleischmann, 2012*a*, his fig. 200). As already recognized by Darwin, the suction traps of the bladderworts belong functionally and architecturally to the most complex structures known from the plant kingdom (Lloyd, 1942; Reifenrath *et al*., 2006; Vincent *et al*., 2011; Adamec, 2011, 2013). Fleischmann (2012, p. 245) wrote: ‘It is hard to imagine how such bladder traps could evolve morphologically in a phylogenetic series, but this evolutionary step is certain to have happened quickly as a key innovation, rather than gradually.’ Both trap types of the *Genlisea–Utricularia* lineage (i.e. eel traps of *Genlisea*, and sucking traps in *Utricularia*) have an early developmental stage in common; they start as peltate (ascidiate) outgrowths (Figs 12D, E and 14C). Jobson *et al*. (2004) presented evidence that the key adaptation in the common ancestor of the *Genlisea–Utricularia* lineage ‘lies in molecular energetic changes that buttressed the mechanisms responsible for the bladderworts’ radical morphological evolution.’ There may be a link between faster reaction kinetics of *Utricularia* traps and a *Utricularia*-specific mutation in COX (cytochrome *c* oxidase) to obtain enough ATP energy (Jobson *et al*., 2004; Laakkonen *et al*., 2006; but see for criticism Adamec, 2011; Król *et al*., 2012).

### ‘Loss-of-root’ hypothesis vs. ‘root–stolon transformation’ hypothesis in the *Genlisea–Utricularia* lineage

It is commonly accepted that *Pinguicula* possesses true roots whereas the *Genlisea–Utricularia* lineage has lost them (Albert *et al*., 2010; Fleischmann, 2012*a*; Carretero-Paulet, 2015*a*, *b*). According to continuum plant morphologists (Brugger and Rutishauser, 1989; Rutishauser and Isler, 2001; Kirchoff *et al*., 2008), the roots were not completely lost in the *Genlisea–Utricularia* lineage. The ancestral roots (as still present in *Pinguicula*) evolved exogenous green appendages that can be called ‘leaves’ again (an idea anticipated by Arber, 1920). Thus, the developmental pathways for roots and shoots were blended (amalgamated) to some degree, perhaps due to co-option of genes usually acting in stems and leaves but not in roots. Arguments in favour of this ‘root–stolon transformation’ hypothesis are as follows. (1) Several *Pinguicula* have roots without caps (e.g. *P. moranensis*). (2) Various Utricularias (e.g. *U. longifolia* and *U. sandersoniii*) have straight stolon tips which look similar (including anatomy) to capless root tips of *Pinguicula*. (3) Although the *Genlisea–Utricularia* lineage has lost several root-specific genes, there are still some left in their vegetative bodies (see paragraph below). (4) Conversion of root meristems to shoot meristems are known from other angiosperms such as *Nasturtium* (Brassicaceae) and *Neottia* (Orchidaceae), pointing to some homology between root and shoot (as discussed by Guédès, 1979). (5) There are common genetic mechanisms that regulate both root and shoot meristems (Friedman *et al*., 2004; Stahl and Simon 2010; Hofhuis *et al*., 2013).

The two seemingly exclusive hypotheses on ‘loss-of-root’ vs. ‘root–stolon transformation’ in the *Genlisea–Utricularia* lineage will probably merge into one if developmental processes and gene actions are emphasized instead of mind-born and arbitrary structural categories (see paragraph below on ‘process morphology and morphospace’).

### Increased mutation rates in Lentibulariaceae may have facilitated the evolution of species richness

The unusual lifestyle of the Lentibulariaceae coincides with genomic peculiarities such as the smallest genomes within angiosperms and extremely high nucleotide substitution rates of their genomes. The two sister genera *Genlisea* and *Utricularia* show the highest DNA mutation rates known amongst all flowering plants (Jobson and Albert, 2002; Jobson *et al*., 2004; Müller *et al*., 2004, 2006; Wicke *et al*., 2013; Carretero-Paulet *et al*., 2015*a*, *b*). Genome and transcriptome analyses were done in three ‘model’ species of Lentibulariaceae: *Genlisea aurea*, *Utricularia gibba* and *U. vulgaris*. Genome size appears highly variable in *Genlisea* and *Utricularia*, and occasionally with miniaturized genomes as low as 1C = 63·4 Mbp, in spite of ancient polyploidization cycles (Ibarra-Laclette *et al*., 2011, 2013; Wicke *et al*., 2013; Veleba *et al*., 2014; Bárta *et al*., 2015). This fast molecular evolution could be connected to the fast speciation and diversification in this group, meaning that genetic shifts are frequent, directly influencing the morphological appearance and therefore the rapid evolution of traps (Jobson and Albert, 2002; Jobson *et al*., 2004, Albert *et al*., 2010; Fleischmann, 2012*a*).

### Developmental genes possibly involved in bauplan deviations in the *Genlisea–Utricularia* lineage

Many developmental genes involved in lentibulariaceous morphology were uncovered within the last years. The genome analyses of *G. aurea*, *U. gibba* and *U. vulgaris* (all of them seemingly rootless) showed the presence of a considerable number of root-specific genes in the vegetative bodies of both *Genlisea* and *Utricularia* (Ibarra-Laclette *et al*., 2011, 2013; Barta *et al*., 2015). Carretero-Paulet *et al*. (2015*a*, *b*) guessed that the specialized bauplan of *U. gibba* may be correlated with the expansion of the *WUS*-like family, whereas the absence of the *WOX5* gene may be correlated with the lack of an obvious root. Bárta *et al.* (2015) wrote: ‘The comparison of the presence or absence of root-associated genes in additional *Utricularia* species will be very useful for understanding the adaptation to an aquatic rootless carnivorous life-style.’ The number of cotyledons (also called ‘cotyledonoids’) in *Utricularia* seedlings is highly variable: between one and 15 or even lacking, depending on the section (Lloyd, 1942; Kumazawa, 1967; Brugger and Rutishauser, 1989; Plachno and Swiatek, 2010). This variability is similar to *laterne* and other mutants known in arabidopsis (Treml *et al*., 2005; Chandler, 2008).

## DISCUSSION AND CONCLUSIONS

This pictorial report emphasizes Lentibulariaceae and Podostemaceae so as to increase our knowledge and understanding of these enigmatic families. Bladderworts and river-weeds are known as morphological misfits because botanists have difficulties in recognizing and delimiting vegetative organs such as foliage leaves, stems and roots. These vegetative organs – as still distinguishable in related flowering plants less deviating from the norm – are blurred (‘fuzzy’) in bladderworts and river-weeds. However, both groups have rather stable (i.e. developmentally robust) floral bauplans.

### Fuzzy concepts in plant morphology and evolutionary developmental biology

Morphological misfits as described for bladderworts and river-weeds transcend traditional structural categories, and cannot be placed fully into one category or the other. In these cases it becomes very difficult, or even impossible, to accept just one name for an organ or appendage. Here a continuum or fuzzy approach could be heuristically fruitful in which structural categories are used as ‘fuzzy sets’, allowing some degree of overlap with related terms (Rutishauser, 1999; Rutishauser *et al*., 2008). A fuzzy approach to plant morphology fits perfectly with the idea, propounded by Darwin (1859), that organisms were formed by gradual transitions between types (Kirchoff *et al*., 2008). This approach is similar to the concepts of partial homology and homeosis that were championed by Sattler (1986, 1988, 1994). Several developmental geneticists seem to be aware of a certain degree of fuzziness in plant development. They used fuzzy concepts such as the ‘leaf–shoot continuum model’ (Sinha, 1999), and ‘mixed shoot–leaf identity’ (Baum and Donoghue, 2002) to describe odd plant structures somewhat intermediate between leaves and shoots (stems) in angiosperms. Eckardt and Baum (2010) wrote: ‘It is now generally accepted that compound leaves express both leaf and shoot properties and that this at least partly reflects ectopic expression of genes related to *STM* in the leaf.’ Tsukaya (2014, p. 214) concluded similarly with respect to a leaf–shoot continuum in angiosperms: ‘Accumulating evidence has suggested that simple leaves, compound leaves, and shoots share common gene regulatory networks (GRNs).’ For example, Tsukaya (2014) provided developmental genetic data on the shoots with green ‘needles’ in asparagus: ‘The phylloclade of *Asparagus asparagoides* is a leaf-like metamorph of the lateral shoot, ectopically expressing some leaf genes.’

### Lack of one-to-one correspondence between structural categories and gene expression

If structural categories do not provide adequate descriptions of plant structure, perhaps it is possible to define structures based on developmental genetics. If there is a one-to-one correspondence between structural units (e.g. roots, leaves and flowers) and the ‘molecular players behind the characters’ (Koentges, 2008), it should be possible to identify the structural units by the expression of well-characterized marker genes. To do this, we need to look for organ identity genes in order to define the structural categories clearly. For example, the *KNOX/ARP* module (as used by Katayama *et al*., 2010, 2013 in Podostemaceae) helps with the determination of the leaf as a determinate unit, and the shoot as an indeterminate module. This approach seems to have promise in the cases where control genes for organ identity have been shown to exist (Kirchoff *et al*., 2008, Langdale and Harrison, 2008; Chormanski and Richards, 2012). Thus, Katayama *et al.* (2010, 2013) identified the ‘leaves’ in Podostemoideae as ‘stem–leaf mixed organs’. This appellation is meant to indicate that these structures have some features of leaves, and some of stems, probably due to their unusual gene expression pattern and the lack of obvious SAMs (Kato, 2013). However, genomic studies in the *Genlisea–Utricularia* lineage may show the opposite (Ibarra-Laclette *et al*., 2011, 2013; Veleba *et al*., 2014; Barta *et al*., 2015; Carretero-Paulet *et al*., 2015*a*, *b*). They pointed to the existence of root-specific genes in the *Genlisea–Utricularia* lineage although their roots were lost (at least seemingly).

The lack of one-to-one correspondence between structural categories and gene expression may arise from the re-use of existing genetic resources in novel contexts. Transcription and signalling factors are often used multiple times in context-specific combinations within an organism (Weiss, 2005; Arthur, 2011). The case studies on bladderworts and river-weeds in the present paper point to plant structures that are difficult to explain by a simple one-to-one correspondence between structure and gene function (Kirchoff *et al*., 2008). Further genetic studies of these organisms will show that at least some of their phenotypic fuzziness results from overlapping or partially indistinct developmental genetic networks.

### Process morphology: morphospace using a set of developmental processes, e.g. in aquatic Utricularias

Wardlaw (1965, p. 371), while pointing to the geologist Charles Lyell, came to the conclusion: ‘Organization is a *continuum* in the physical world. Organization is also a *continuum* in the ontogenesis and reproduction of the individual organism and in the phyletic line of which it is a component.’ Therefore, we may ask as a more specific question with respect to the bladderworts and river-weeds having unusual morphologies: is the recognition of developmental processes (e.g. branching patterns and growth patterns) more important than proper definition of structural units, i.e. plant organs such as roots, stems and leaves? Process morphology (or dynamic morphology) *sensu* Sattler (1994) and Jeune *et al*. (2006) allows us to dispense with all structural categories and characterize phenotypes by sets of developmental processes. The living forms we perceive and conceive of in the realms of multicellular organisms (animals, plants, fungi) ‘are only a small subset of the possible forms we could imagine’ (Minelli, 2015*c*). The theoretical morphospace includes all possible process combinations for seed plants, whereas the empirical morphospace contains only those process combinations that are realized in nature (Niklas, 1997, p. 215). Each axis of the morphospace corresponds to a variable that describes some developmental processes of an organism, or its parts. The use of a single morphospace to which gene expression can be annotated is appealing, especially so since its use would remove most, if not all of the terminological problems described above. Unlike rigid categorical vocabularies, process morphology should allow better hypotheses about the ‘molecular players behind the characters’ (Koentges, 2008). Thus, Sattler and Rutishauser (1990), Jeune *et al*. (2006) and Kirchoff *et al.* (2008) represented the vegetative bodies of aquatic Utricularias (e.g. *U. foliosa*) and other morphological misfits as combinations of developmental processes using multivariate statistical analyses. Plant organs (e.g. watershoots, leaves or bracts of *U. foliosa*) are identified in the morphospace as specific process combinations. No doubt the use of process combinations to describe plant structures makes communication among scientists difficult. Nevertheless, one of the great strengths of this approach is that the categorical terms (e.g. ‘leaf’ and ‘shoot’) serve only as placeholders for combinations of developmental processes that locate the organs in the morphospace. Gene expression patterns of the ‘model’ bladderworts (such as *U. vulgaris* and *U. gibba*) and related Genliseas may finally be annotated to the morphospace by associating the expression pattern with the combination of processes that are found in the part in which the gene is expressed (Kirchoff *et al*., 2008; Chormansky and Richards, 2012; Carretero-Paulet *et al*., 2015*a*, *b*).

### Adaptive value of bauplan features vs. patio ludens

While introducing ‘adaptive walks in aquatic and terrestrial landscapes’ Niklas (1997) assigned a relative fitness to each phenotype in a morphospace, although this task is far from simple. Niklas (1997, p. 218) explained why: ‘The phenotypic plasticity of plants appears to be extremely high in comparison with that of most animals.’ According to Willis (1914), the unique features in which the various genera and species in Podostemaceae differ from one another cannot be explained as simply adaptational. This hypothesis was taken over by van Steenis (1981) who proposed the concept of ‘patio ludens’ (evolutionary playground). Plants in certain habitats evolved forms that are difficult to explain by adaptive occupation of species-specific ecological niches. According to Willis and van Steenis, the river-weeds evolved, in the more or less homogenous environments of waterfalls and river-rapids, new and fanciful mutants that did not (yet) become erased by natural selection. Some of these mutants, perhaps resulting from saltational evolution, became stabilized, leading to new species (see also Arber, 1920; Rutishauser, 1997). As expressed by Wardlaw (1965, p. 392ff) ‘patio ludens’ ideas are difficult to confirm**,** although it is also difficult to support the opposite, i.e. to assign a relative fitness (adaptive value) to each phenotype. Patio ludens coincides to some degree with what is labelled as ‘evolutionary freedom’ by Minelli (2015*b*, p. 335).

### Physiological adaptations

With respect to bladderworts and river-weeds, one should keep in mind that physiological parameters such as seedling establishment, mineral nutrient uptake, photosynthesis, mitochondrial respiration and sexual vs. clonal reproduction may be more important than vegetative bauplan characters for successful speciation (survival of the fittest). Both families exhibit extreme physiological adaptations with respect to habitats. The unfavourable environmental conditions (including nutrient-poor habitats) of Lentibulariaceae and Podostemaceae may have been counterbalanced by efficient carnivory and symbiosis with cyanobacteria, respectively (Jäger and Grubert, 2000; Mohan Ram and Sehgal, 2001; Jobson *et al*., 2004; Laakkonen *et al*., 2006; Vincent *et al*., 2011; Król *et al*., 2012; Adamec 2013; Wicke *et al*., 2013; Plachno *et al.*, 2014*a*).

### Hopeful monsters and saltational evolution

Until recently, most evolutionary biologists were convinced that gradualism (cf. Darwin, 1859) reflects the most frequent mode of evolution. Drastic (saltational) evolutionary innovations of new phenotypes were regarded as highly improbable by most evolutionary biologists (Niklas, 1997; Arthur, 2011). However, in some cases, profound (saltational) changes may have occurred within one or a few generations. Organisms with a profound mutant phenotype that have the potential to establish a new evolutionary lineage have been termed ‘hopeful monsters’ (Goldschmidt, 1952; Bateman and DiMichele, 2002; Theissen, 2006, 2009). Recent discoveries in genomics, epigenetics and evo-devo increased the credibility of saltational hypotheses. For example, Masel and Siegal (2009) gave hopeful monsters a new chance to survive. Their reasoning is based on new evolutionary concepts such as developmental robustness and evolvability of living systems (including evolutionary capacitance, genetic assimilation *sensu* Waddington, 1953). Masel and Siegal (2009) wrote: ‘Evolutionary capacitance, whether evolved or intrinsic, reopens the idea, introduced by Richard Goldschmidt, of “hopeful monsters” in evolution … A single capacitor mutation could have a large effect by phenotypically revealing a large number of pre-existing variants, each of small effect … Large-effect mutations that participate in adaptation might simply arise in genes encoding capacitors that normally provide robustness to many small-effect mutations at other sites.’ Thus, hopeful monsters and saltational evolution get a revival as valuable biological concepts. Far from being mutually exclusive scenarios, both gradualism and saltationism are required to explain the complexity and diversity of life on Earth (Theissen, 2006, 2009; Minelli, 2015*a*).

Bladderworts and river-weeds provide nice examples of hopeful monsters that are ‘here to stay’ (Theissen, 2009). For example, saltational evolution may have amplified the morphological diversity in Podostemaceae – Tristichoideae (Figs 2–5), especially in the *Dalzellia–Indotristicha* lineage, with *Indodalzellia* as their sister genus (Koi *et al*., 2012; Fujinami and Imaichi, 2015).

### Floral vs. vegetative bauplans in angiosperms

In both eudicot families, Lentibulariaceae and Podostemaceae, floral and vegetative bauplans can be distinguished. Their floral bauplans appear to be rather stable (robust), reflecting their affiliation with euasterids (order Lamiales) and eurosids (order Malpighiales), respectively. When these two families are labelled as morphological misfits, it is due to their loss of important characters of the CRS bauplan as typical for most angiosperms (Rutishauser and Isler, 2001; Rutishauser *et al*., 2008). Loss of the CRS bauplan does not mean that the vegetative bodies of bladderworts and river-weeds tend to be chaotic. They just took over new rules of forms, i.e. new patterns of body syntax (Minelli, 2015*c*), leading to new vegetative bauplans that are specific for the various subgroups of, for example, *Utricularia* (Figs 9 and 10G).

Molecular genetic work will soon provide deeper insight into the developmental switches responsible for the (partial) loss of the CRS bauplan in bladderworts and river-weeds, as compared with more typical seed plants. Understanding what developmental patterns are followed in morphological misfits is a necessary prerequisite to discover the developmental genetic alterations that led to the establishment of these odd angiosperms having unusual morphologies.

### Is species diversity in Lentibulariaceae and Podostemaceae facilitated by the loss of developmental robustness of their vegetative bodies?

Biological systems are robust if they continue to function in the face of genetical perturbations and environmental change (Wagner, 2005, 2008; Ehrenreich and Pfennig, 2016). Developmental robustness is identical, or nearly so, to Waddington’s (1953) concept of ‘developmental canalization’: an organismal feature is canalized if its embryonic development is insensitive to variation in the environment or its genes. The switches to new and unique vegetative bauplans in Lentibulariaceae and Podostemaceae probably faciliated rather than prevented the evolution of species diversity. *Utricularia* (including *Polypompholyx*) contains 230 species, being by far the largest genus of all carnivorous angiosperms (Veleba *et al*., 2014). The river-weeds (Podostemaceae) with a total of approx. 54 genera and approx. 310 species are by far the largest family of truly aquatic angiosperms (Cook and Rutishauser, 2007; Kato, 2012, 2013). The relatively high number of genera for only slightly more than 300 species in Podostemaceae is difficult to explain. Kato (2012) added as a possible explanation: ‘Large-gapped body plans evolved in an apparently uniform environment.’ Other flowering plant families containing morphological misfits such as the Gesneriaceae (with one-leaf plants in *Monophyllaea* and *Streptocarpus*) will have to be checked (Möller and Cronk, 2001; Ayano *et al*., 2005; Harrison *et al*., 2005): were these odd angiosperms with rather stable floral bauplans but unusual vegetative morphologies also able to produce more species (and genera) as compared with their next-related sister groups with CRS bauplan?
